# Supplementary material for: Asatone and Isoasatone A Against Spodoptera litura Fab. by Acting on Cytochrome P450 Monoxygenases and Glutathione Transferases
Source: Molecules. 2019 Oct 31;24(21):3940. doi: 10.3390/molecules24213940 (PMC6864857; doi:10.3390/molecules24213940)
Supplement: Supplementary file 1 [file molecules-24-03940-s001.pdf]

Suppl. table 1 Primers used in this study

Gene Name	Forward primer (5'---3')	Reverse primer (5'---3')	size (bp)	Gene bank
**SlAce1**	AGGAACCGAAATGCTTTGGA	GGATTCAAAGGCTCCCCGAA	206	KY130418
**SlAce2**	GCTCCGGGAAATATGGGCTT	TCAGCGGCAAGAAGACTACTG	296	KY130419
**CYP6AB14**	GTTCGTGTATCTTCCCTTCG	ATAACGGAGCGTGGAGGGA	135	JF829002.1
**CYP321A7**	GCTATTCATCACGACCCA	CCAGCCTGCACTTGTAAT	161	MF802804.1
**CYP6B47**	AAGTGGCAAGGGACTACCAA	ATCCGATGCAGTTTCGCTGT	213	JN862817.1
**CYP6B58**	CTGTATCGGCATGCGGTTTG	GGTGAGGTTCCACCTCCAAG	119	GU263828.1
**CYP321B1**	CTTAGCTGGCATTGACCCCA	GCATCATACGTCAGCTTGCC	141	GU263829.1
**CYP9A39**	CCTCTACCAGAGCTTCCCTG	TCAGTGTAGAGCGCATGTCC	221	GQ465040.1
**SlGSTe1**	CCCCACTAAAGAACAAACCG	GATGATACACTAGCTGCCAG	132	AY506545.1
**SlGSTo1**	CGCTATGAGGTTCTGTCC	CCTTTAGGGCTGTAGTTG	129	HQ667939.1
**Actin**	CCACGAGACCACTTACAAC	GCCAGAGCAGTGATTTCC	141	NC_036215

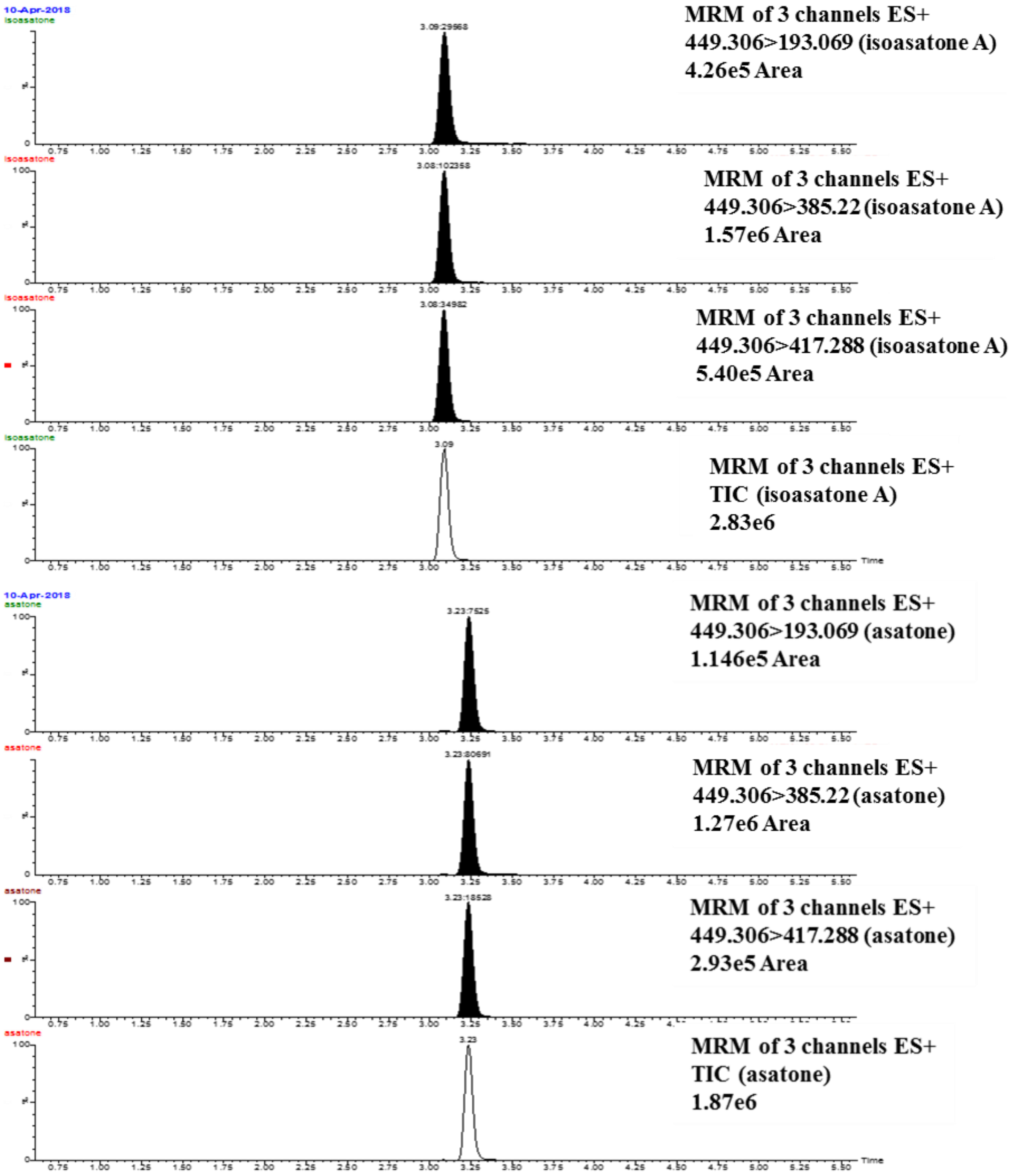

Suppl. Fig. S1. Established MRM model for asatone and isoasatone A

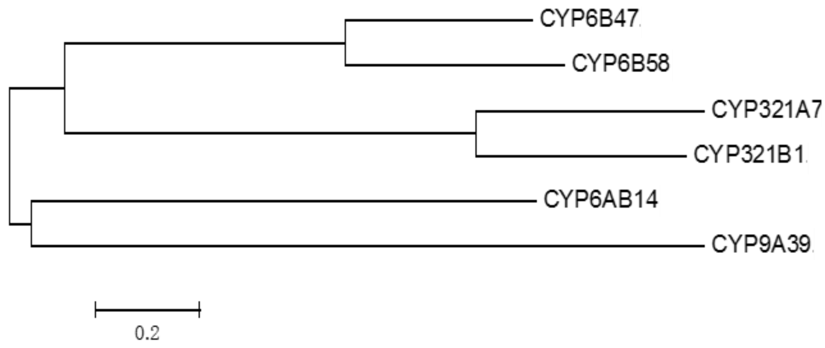

Suppl. Fig. S2. Phylogenetic analysis of selected P450s from *Spodoptera litura*. The phylogenetic tree was constructed with MEGA 6.06 software using Neighbour-joining method and 1000 bootstrap replications. The scale bar indicates 0.2 amino acid substitutions per site.
